# The complete chloroplast genome of *Rudbeckia hirta* L. (Asteraceae) and its phylogenetic analysis

**DOI:** 10.1080/23802359.2026.2742618

**Published:** 2026-10-10

**Authors:** Chenfeng Gao, Xiaohan Wang, Yanyan Zhang, Qingxiang Meng, Hongqin Li

**Affiliations:** ^a^ College of Pharmacy, Heze University, Heze, PR China; ^b^ College of Pharmacy, Heze Biomedicine Vocational College, Heze, PR China

**Keywords:** Asteraceae, chloroplast genome, Heliantheae, phylogenetic analysis, *Rudbeckia hirta*

## Abstract

*Rudbeckia hirta*, a widely cultivated North American ornamental, has pharmacological value due to its secondary metabolites. Herein, we assembled and characterized its first complete chloroplast genome from high-throughput sequencing reads. The 151,743 bp genome contains 127 genes, including 85 protein-coding, 34 tRNA and 8 rRNA genes. The long single copy region has three nested inversions: an 83,300 bp large inversion enclosing an 18,500 bp medium inversion with a 3,700 bp small inversion inside. Phylogenetic analysis robustly clustered *R. hirta* with *R. laciniata var. laciniata* (bootstrap 100%). This genomic resource facilitates future research on its phylogeny, genetic diversity and potential exploitation.

## Introduction

*Rudbeckia hirta* L. 1753, commonly known as black-eyed Susan, belongs to the family Asteraceae and is widely cultivated as an herbaceous ornamental. Besides their horticultural uses, *Rudbeckia* species have been used in traditional medicine and investigated for anti-inflammatory, antimicrobial, antioxidant, immunomodulatory, and other biological activities (Vardeman et al. 2022). Phytochemical analyses of *R. hirta* flowers identified phenolic acids, phenolic acid esters, flavonol glycosides, and other bioactive metabolites (Michael et al. 2014), and recent metabolomic studies detected sesquiterpene lactones, polyphenolic acids, flavonoids, amino acids, and fatty acids (Burlec et al. 2023). These findings suggest that *R. hirta* may be a useful resource for further phytochemical and pharmacological research.

Despite its medicinal potential, detailed genomic studies of *R. hirta* remain limited. Although several nuclear and chloroplast genome markers (e.g. *matK* and *rbcL*) are available in GenBank, a complete chloroplast genome of *R. hirta* has not been reported to date. In recent years, chloroplast genomes have been widely used in plant evolutionary studies because of their conserved organization and relatively stable inheritance patterns. Here, we assembled, annotated, and analyzed the complete chloroplast genome of *R. hirta*. These data are expected to clarify its phylogenetic position and provide resources for future genetic and applied research.

## Materials and methods

Fresh *R. hirta* leaves for chloroplast genome sequencing were sampled in Peony District, Heze City, Shandong Province, China (35°18′55.37ʺN, 115°30′38.77ʺE) (Figure 1). A voucher specimen was deposited in the Herbarium of Heze University (contact: Hongqin Li, 463056627@qq.com) with voucher number HZ220812.

**Figure 1.**
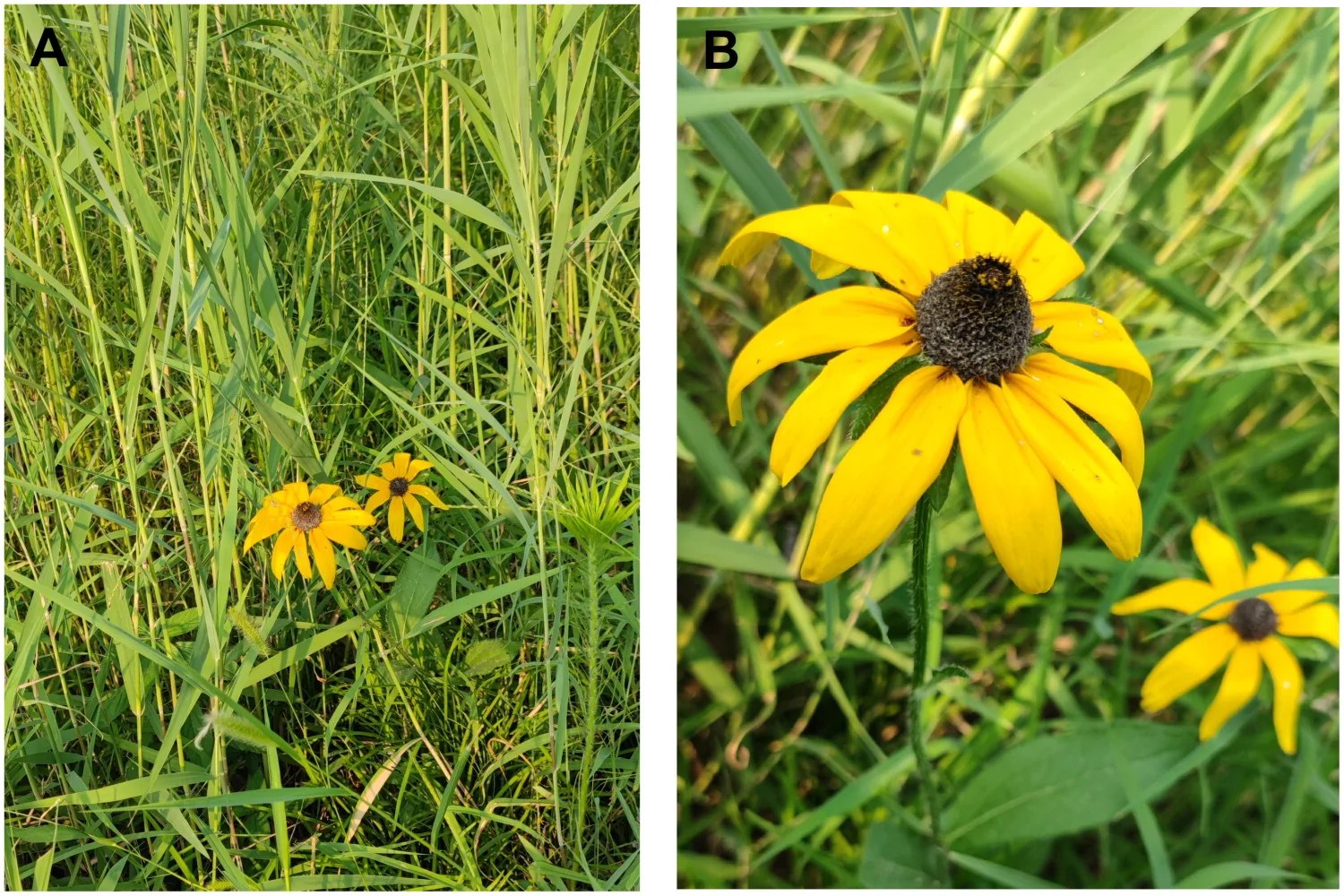
Field photograph of *Rudbeckia hirta*. Location: 35°18′55.37ʺN, 115°30′38.77ʺE (photographer: Hongqin Li). The plant has rough pubescence, alternate serrated leaves, long-pedunculate terminal capitula, yellow ray florets, and a dark brown to black central disk. (A) Panoramic view of the whole plant. (B) Top-down view of the capitulum.

Fresh, healthy young leaves of *R. hirta* were collected in the field, immediately frozen in liquid nitrogen, and stored at −80 °C until DNA extraction. Total genomic DNA was then isolated using the Plant Genomic DNA Kit (Tiangen Biotech, Beijing, China). The extracted DNA was fragmented to approximately 300 bp for short-insert library construction, and the libraries were sequenced on the Illumina NovaSeq 6000 platform at Wuhan Benagen Technology Company Limited (Wuhan, China). Raw reads were trimmed using Trimmomatic (v0.35) (Bolger et al. 2014) with default parameters to remove adapter sequences and low-quality bases. After quality control, the clean reads were assembled *de novo* with GetOrganelle (v1.7.1) (Jin et al. 2020). The complete chloroplast genome was annotated using the online tool CPGAVAS2 (http://47.96.249.172:16019/analyzer/annotate) (Shi et al. 2019), and the annotations were manually corrected using Apollo (Dunn et al. 2019). The annotated chloroplast genome was deposited in GenBank. A circular chloroplast genome map was generated using CPGview (http://47.96.249.172:16085/cpgview/drawmap) (Liu et al. 2023). Whole-genome synteny was examined using Mauve (v2.4.0) to compare the *R. hirta* chloroplast genome with those of closely related Asteraceae taxa. To verify the inversion breakpoints, we mapped raw reads to the assembled chloroplast genome, and we inspected the alignments in Tablet (v1.21.02.08).

For phylogenetic analysis, complete chloroplast genome sequences from 37 related species, including *Codonopsis pilosula* and *Campanula takesimana* as outgroups, were retrieved from GenBank, and we combined them with the *R. hirta* sequence from this study to form a dataset of 38 taxa. Whole chloroplast genomes were aligned using MAFFT with default parameters (Katoh and Standley 2016). A maximum-likelihood (ML) phylogenetic tree was inferred using IQ-TREE (v2.0) (Nguyen et al. 2015) under the best-fit model TVM + F + G4, with 1000 bootstrap replicates. The resulting ML tree was displayed and refined using the online tool iTOL (v7.6) (Letunic and Bork 2024).

## Results

The chloroplast genome of *R. hirta* was assembled into a circular DNA molecule of 151,743 bp, with a typical quadripartite structure (Figure 2). Sequencing coverage of the assembled chloroplast genome was high, with an average depth of 716.54× and a range of 4× to 1317× (Figure S1), supporting the completeness and reliability of the assembly. The genome comprised a large single-copy (LSC) region of 83,330 bp, a small single-copy (SSC) region of 18,405 bp, and two inverted repeat (IR) regions of 25,004 bp each (Figure 2). The overall GC content was 37.63%, and the GC contents of the LSC, SSC, and IR regions were 35.77%, 31.24%, and 43.07%, respectively. The higher GC content in the IR regions is likely related to their GC-rich rRNA genes. Comparison of the *R. hirta* and *Barnadesia caryophylla* chloroplast genomes using a dot plot and Mauve synteny analysis identified a triple inversion in the LSC, comprising three nested inversions (∼83,300 bp, ∼18,500 bp, and ∼3700 bp; Figure S2 and Figure S3A). The inversion breakpoints were validated by read mapping results (Figure S3B–E).

**Figure 2.**
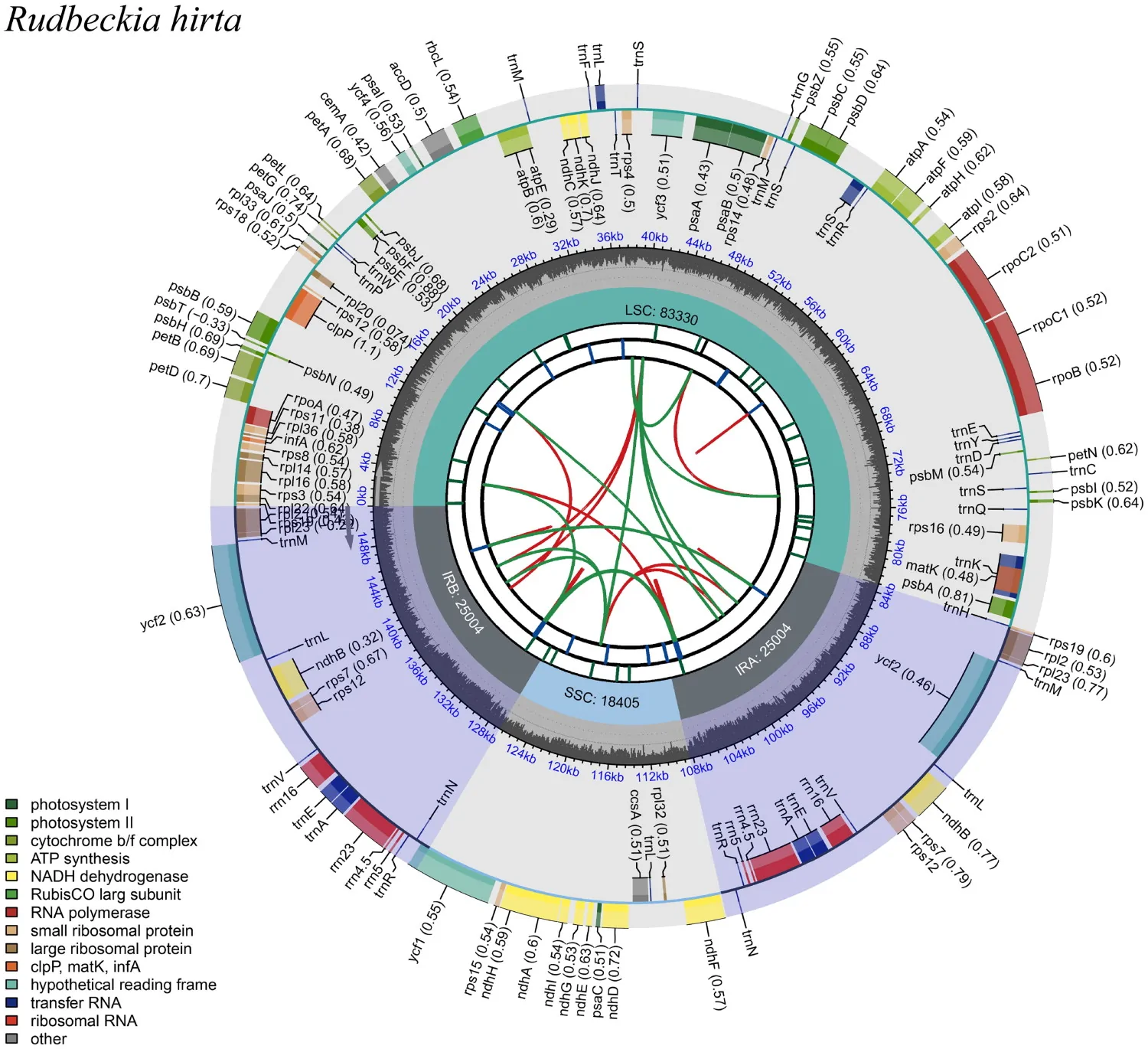
The complete chloroplast genome map of *Rudbeckia hirta*. The map contains six tracks. From the center outward, the first track shows the dispersed repeats. The second track displays long tandem repeats as short blue bars. The third track illustrates SSRs as short bars with various colors. The small single-copy (SSC), inverted repeat (IRA and IRB), and large single-copy (LSC) regions are represented in the fourth track. The GC content along the genome is visualized in the fifth track. The genes are shown in the sixth track, with genes from different functional groups color-coded. Genes transcribed in a clockwise direction are located on the outside of the map, while genes transcribed counterclockwise are shown on the inside.

Annotation of the *R. hirta* chloroplast genome identified 127 genes, comprising 85 protein-coding genes (PCGs), 34 tRNA genes, and eight rRNA genes. These 34 tRNA loci correspond to 25 unique tRNA genes. Relative to typical angiosperm chloroplast genomes with 30 unique tRNA genes, the six missing tRNA genes are *trnV-UAC*, *trnT-GGU*, *trnfM*-*CAU*, *trnG*-*UCC*, *trnH*-*GUG*, and *trnI*-*CAU* (all located in the LSC region). However, *trnS*-*CGA* occurs in *R. hirta* but is absent from the standard ∼30 tRNA core set in angiosperms (Alkatib et al. 2012). Genes duplicated in the IR regions included seven PCGs (*ndhB*, *rpl2*, *rpl23*, *rps7*, *rps12*, *rps19*, and *ycf2*), seven tRNA genes (*trnA-UGC*, *trnE-UUC*, *trnL-CAA*, *trnM-CAU*, *trnN-GUU*, *trnR-ACG*, and *trnV-GAC*), and four rRNA genes (*rrn16S*, *rrn23S*, *rrn4.5S*, and *rrn5S*). Introns were present in both PCGs and tRNA genes. Among the PCGs, *atpF*, *ndhA*, *ndhB*, *petB*, *petD*, *rpl2*, *rpl16*, *rpoC1*, and *rps16* each had one intron, whereas *clpP* and *ycf3* each had two introns (Figure S4). Five unique tRNA genes, namely *trnA-UGC*, *trnE-UUC*, *trnK-UUU*, *trnL-UAA*, and *trnS-CGA*, each contained one intron. In addition, *rps12* was identified as a trans-spliced gene. The structures of cis-/trans-spliced PCGs are presented in Figures S4 and S5.

The ML tree inferred from chloroplast genome data had high bootstrap support (≥80%) for all major clades (Figure 3, Figure S6). In this analysis, *R. hirta* and *R. laciniata* var. *laciniata* formed a monophyletic group. This clade had maximum bootstrap support (100%), confirming a close evolutionary relationship between them. *Tithonia* and *Helianthus* also formed a monophyletic clade (bootstrap support = 100%). This clade grouped with *Echinacea* to form a larger clade (bootstrap support = 82%). The larger clade subsequently formed a sister group with *Ambrosia*, and then formed a sister relationship with *Rudbeckia* (bootstrap support = 99%).

**Figure 3.**
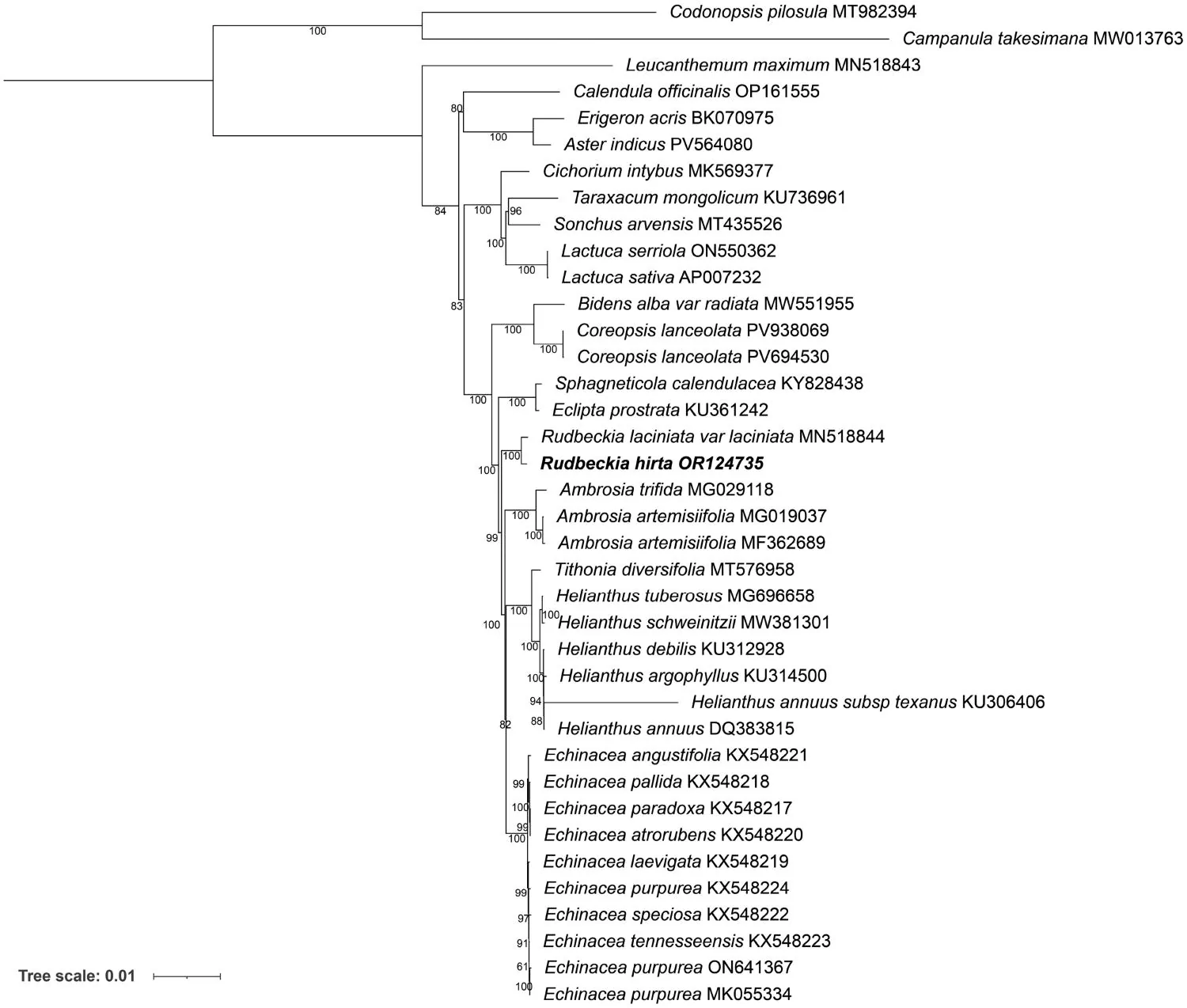
Maximum-likelihood phylogeny of *Rudbeckia hirta* and its close relatives based on complete chloroplast genome sequences. Bootstrap values based on 1000 replicates are shown at the nodes. The dataset included 37 related species downloaded from GenBank. They were: *Codonopsis pilosula* MT982394 (outgroup), *Campanula takesimana* MW013763 (outgroup) (Park et al. 2021), *Leucanthemum maximum* MN518843 (Chen et al. 2019), *Calendula officinalis* OP161555 (Zhang et al. 2024), *Erigeron acris* BK070975 (Abdullah et al. 2025), *Aster indicus* PV564080, *Cichorium intybus* MK569377 (Yang et al. 2019), *Taraxacum mongolicum* KU736961 (Kim et al. 2016), *Sonchus arvensis* MT435526 (Abdalla Masana et al. 2020), *Lactuca serriola* ON550362, *Lactuca sativa* AP007232, *Bidens alba* var. *radiata* MW551955 (Wu et al. 2022), *Coreopsis lanceolata* PV938069, *Coreopsis lanceolata* PV694530 (Wan et al. 2025), *Sphagneticola calendulacea* KY828438 (Ng et al. 2018), *Eclipta prostrata* KU361242 (Park et al. 2016), *R. laciniata* var. *laciniata* MN518844, *Ambrosia trifida* MG029118 (Sablok et al. 2019), *Ambrosia artemisiifolia* MG019037 (Amiryousefi et al. 2017), *Ambrosia artemisiifolia* MF362689 (Nagy et al. 2017), *Tithonia diversifolia* MT576958, *Helianthus tuberosus* MG696658, *Helianthus schweinitzii* MW381301, *Helianthus debilis* KU312928, *Helianthus argophyllus* KU314500, *Helianthus annuus* subsp. *texanus* KU306406, *Helianthus annuus* DQ383815, *Echinacea angustifolia* KX548221 (Zhang et al. 2017), *Echinacea pallida* KX548218 (Zhang et al. 2017), *Echinacea paradoxa* KX548217 (Zhang et al. 2017), *Echinacea atrorubens* KX548220 (Zhang et al. 2017), *Echinacea laevigata* KX548219 (Zhang et al. 2017), *Echinacea purpurea* KX548224 (Zhang et al. 2017), *Echinacea speciosa* KX548222 (Zhang et al. 2017), *Echinacea tennesseensis* KX548223 (Zhang et al. 2017), *Echinacea purpurea* ON641367, *Echinacea purpurea* MK055334, and the *R. hirta* (OR124735) generated in this study was labeled in bold font.

## Conclusions and discussion

This study describes the complete chloroplast genome of *R. hirta*, which has a typical quadripartite structure, measures 151,743 bp and contains 127 annotated genes. *R. hirta* has only 25 unique tRNA genes, with the same composition as *R. laciniata*. This differs from the usual angiosperm complement of ∼30 tRNA genes, indicating that a reduced tRNA repertoire may be a genus-level trait in *Rudbeckia*. The loss of *trnT-GGU* in *R. hirta* agrees with previous findings in core Asteraceae, where this gene is often pseudogenized or lost (Abdullah et al. 2021), suggesting a recurrent evolutionary event in this family. The genome size, gene composition, GC content distribution, and distribution of intron-containing genes generally match the conserved characteristics of Asteraceae chloroplast genomes (Mahai et al. 2024; Nguyen et al. 2024a). Although most non-basal Asteraceae species share a canonical double inversion (Timme et al. 2007; Kumar et al. 2009; Salih et al. 2017; Loeuille et al. 2021; Li et al. 2025; Wan et al. 2025; Table S1), *R. hirta* shows a more complex triple inversion in the LSC. A similar triple inversion has been reported in *Cosmos sulphureus* (Chen et al. 2026). Related structural rearrangements, including IR expansions, are also found in other angiosperm families (e.g. Onagraceae; Nguyen et al. 2024b), indicating that inversions and genomic rearrangements repeatedly contribute to chloroplast genome diversity across distantly related lineages.

Phylogenetic analysis confirmed that *R. hirta* and *R. laciniata* formed a monophyletic clade (100% BS). *Echinacea* was sister to the *Helianthus* + *Tithonia* clade (82% BS), which conflicts with its classical placement in subtribe Zinniinae (Robinson 1981; Urbatsch and Jansen 1995; Urbatsch et al. 2000). This relationship suggests that subtribal boundaries within Heliantheae may need reevaluation. In contrast, *Rudbeckia* was clearly distinct from the *Echinacea*-containing clade (99% BS), consistent with the traditional view. These phylogenetic relationships require further testing with nuclear markers and broader sampling.

## Supplementary Material

Supplemental Material

## Data Availability

The complete chloroplast genome sequence of *R. hirta* in this study has been submitted to the NCBI database under the accession number OR124735. The associated BioProject, SRA, and Bio-Sample numbers are PRJNA1146888, SRR38968011, and SAMN60586596, respectively.
