# Supplementary material for: The complete chloroplast genome of Rudbeckia hirta L. (Asteraceae) and its phylogenetic analysis
Source: Mitochondrial DNA B Resour. 2026 Oct 10;11(11):1300–5. doi: 10.1080/23802359.2026.2742618 (PMC13656687; doi:10.1080/23802359.2026.2742618)
Supplement: Supplementary file 1 [file TMDN_A_2742618_SM6195.docx]

**Figure S1. The coverage depth of the chloroplast genome assembly of *Rudbeckia hirta.***

**Figure S2. Dotplot map comparing the chloroplast genomes of *Barnadesia caryophylla* (OM892817.1, X-axis) and *Rudbeckia hirta* (OR124735, Y-axis).**

**A**

**B**

**C**

**D**

**E**

**Figure S3.** **Whole-plastome synteny analysis and inversion breakpoint validation in *Rudbeckia hirta*.**

(A) Mauve alignment of the *R*. *hirta* chloroplast genome with those of multiple closely related Asteraceae taxa. The Mauve alignment clearly revealed the presence of three distinct inversions in the LSC region of *R*. *hirta*: a large inversion (~83,300 bp, highlighted with a red box) nested with a medium inversion (~18,500 bp, highlighted with a blue box), which in turn enclosed a small inversion (~3,700 bp, highlighted with a rose box).

(B–E): Tablet screenshots validated the five inversion breakpoints at single-nucleotide resolution, which were (B) 52,224 bp; (C) 70,730 bp; (D) 74,446 bp; and (E) 83,321 bp. Read sequencing depth at each junction was similar to the genome-wide average sequencing depth(716.54×). All breakpoints were labelled with red numbers.

**Figure S4. Schematic map of the cis-splicing genes in the chloroplast genome of *Rudbeckia hirta*.** Exons and introns are shown in black and white, respectively. The arrow indicates the sense direction of the gene.

**Figure S5. Schematic map of the trans-splicing gene *rps*12 in the chloroplast genome of *Rudbeckia hirta*.**

**Figure S6. Maximum-likelihood phylogeny of *Rudbeckia hirta* and its close relatives based on complete chloroplast genome sequences**

**Table S1. Inversion patterns and sizes in Asteraceae chloroplast genomes**

| Genus/Species | Genome size (bp) | Inversion pattern | Inversion sizes | Reference |
| --- | --- | --- | --- | --- |
| *Helianthus annuus* | 151,104 | Double inversion (nested) | 22.8 kb + 3.3 kb | Timme et al. 2007 |
| *Lactuca sativa* | 152,772 | Double inversion (nested) | 22.8 kb + 3.3 kb | Timme et al. 2007 |
| *Gaillardia pulchella** | 151778 | Double inversion (nested) | ~18.6 kb* + ~3.3 kb* | Li et al. 2025 |
| *Coreopsis lanceolata* | 150,448 | Double inversion (nested) | ~18.8 kb + ~ 2.7 kb | Wan et al. 2025 |
| *Parthenium argentatum* | 152,803 | Double inversion (nested) | 22,890 bp + 3,364 bp | Kumar et al. 2009 |
| *Aldama anchusifolia* | 151,330 | Double inversion (nested) | 22,360 bp + 3,298 bp | Loeuille et al. 2021 |
| *Taraxacum obtusifrons* O978 / *T. stridulum* S3 | 151,322 | hemi-nested inversions | 21,737 bp + 2,543 bp | Salih et al. 2017 |
| *Taraxacum amplum* A978 | 151,349 | hemi-nested inversions | 21,711 bp + 2,542 bp | Salih et al. 2017 |

* Inversion sizes were determined in this study, but not specified by Li et al. (2025).
